# STC1 promotes colorectal cancer invasion and migration by regulating M2 macrophage polarization via TGF-β1/Smad signaling pathway

**DOI:** 10.3389/fmed.2026.1775478

**Published:** 2026-05-05

**Authors:** Xin Chen, Youying Lai, Yichen Zhou, Haoyue Wang, Junkai Wen, Yi Zou, Haotian Cui, Wanli Deng, Xinwen Ma, Xueqing Hu, Yanbo Zhang

**Affiliations:** 1 Shuguang Hospital Affiliated to Shanghai University of Traditional Chinese Medicine, Shanghai, China; 2 Department of Medical Oncology, Shuguang Hospital, Shanghai University of Traditional Chinese Medicine, Shanghai, China; 3 Clinical Research Unit, Shuguang Hospital Affiliated to Shanghai University of Traditional Chinese Medicine, Shanghai, China

**Keywords:** colorectal cancer, STC1, macrophages, TGF-β1/Smad, invasion and migration

## Abstract

**Objectives:**

Colorectal cancer (CRC) mortality is largely attributable to invasion, migration, and an immunosuppressive tumor microenvironment. We examined the clinical relevance and mechanistic function of stanniocalcin-1 (STC1) in CRC, with emphasis on macrophage polarization.

**Methods:**

STC1 expression patterns, prognostic value, and associated pathways were analyzed in TCGA and GEO CRC datasets, followed by KEGG enrichment. STC1 was silenced in HCT116 and SW620 cells using siRNA. Wound-healing and Transwell migration/invasion assays, immunofluorescence, qRT-PCR, ELISA and Western blotting were performed to evaluate metastatic phenotypes, epithelial–mesenchymal transition (EMT), and TGF-β1/Smad signaling. Correlations between STC1 and immune infiltration were assessed in TCGA. Conditioned-medium and co-culture experiments, together with flow cytometry analysis of M2-associated surface markers were used to determine the impact of STC1 on macrophage polarization and the reciprocal effects of polarized macrophages on CRC cell behavior.

**Results:**

STC1 was significantly upregulated in CRC, and high STC1 expression was associated with worse overall and disease-free survival. STC1 knockdown markedly reduced CRC cell migration and invasion and attenuated EMT, as evidenced by increased E-cadherin and decreased N-cadherin/vimentin. Bioinformatic and experimental analyses indicated that STC1 promotes CRC progression via activation of the TGF-β1/Smad pathway. STC1 levels correlated positively with macrophage infiltration in CRC tissues. *In vitro*, STC1 promoted M2 macrophage polarization, while M2 macrophages enhanced EMT and metastatic traits of CRC cells, supporting a pro-tumor positive-feedback loop.

**Conclusion:**

STC1 facilitates CRC invasion and migration by activating TGF-β1/Smad signaling and driving M2 macrophage polarization, suggesting its utility as a prognostic biomarker and therapeutic target.

## 1 Introduction

Malignancies represent a major and growing global health burden. Colorectal cancer (CRC) ranks among the most frequently diagnosed cancers worldwide and remains a leading cause of cancer-related death (1). Despite improvements in multimodal therapies, including neoadjuvant chemotherapy, the overall 5-year survival rate for CRC has not increased substantially (2). Therefore, uncovering key molecular factors and immune-related mechanisms that drive CRC is essential for developing more effective targeted therapies.

The tumor microenvironment (TME) is a principal determinant of CRC invasion and migration (3, 4). Cellular heterogeneity within the TME provides multiple therapeutic entry points. Among these components, macrophages exhibit pronounced plasticity and can polarize into classically activated pro-inflammatory (M1) or alternatively activated immunosuppressive (M2) phenotypes in response to local signals, thereby shaping tumor evolution (5–7). M2-polarized macrophages are widely recognized as pro-tumorigenic, promoting angiogenesis, extracellular-matrix remodeling, metastatic dissemination, and immune evasion (8). In CRC, expansion of M2 macrophages in the TME has been consistently associated with adverse clinical outcomes (9, 10). Thus, strategies that disrupt M2 polarization or reprogram macrophage states have emerged as promising approaches for CRC immunotherapy.

Stanniocalcin-1 (STC1), a glycoprotein hormone located on chromosome 8p21.2, was originally characterized as a regulator of calcium/phosphate homeostasis but is increasingly implicated in cancer biology (11–13). Multi-cancer studies indicate that STC1 can promote malignant traits through distinct pathways, including IL-6/JAK2/STAT3 activation in gastric cancer (14); cancer-associated fibroblast-mediated positive feedback in breast cancer (15); Nrf2-driven metabolic reprogramming in prostate cancer (16); and FOXC2/ITGB6 signaling in ovarian cancer (17); In melanoma, STC1 enhances YAP nuclear translocation and recruits M2 macrophages to drive angiogenesis and migration (18). Notably, STC1 may exert context-dependent effects, showing tumor-suppressive activity in some cancers (19), However, whether STC1 regulates immune remodeling and metastatic progression in CRC, and the underlying molecular circuitry, remain insufficiently defined.

Here, by integrating bioinformatic analyses with *in vitro* validation, we demonstrate that STC1 accelerates CRC invasion and migration by activating the TGF-β1/Smad axis and promoting macrophage polarization toward the M2 phenotype. We further show that M2 macrophages reinforce CRC EMT and metastatic traits, forming an STC1-dependent positive feedback loop. These findings identify STC1 as a driver of immune-assisted migration in CRC and suggest a potential therapeutic avenue to interrupt TME-mediated tumor progression.

## 2 Materials and methods

### 2.1 Data collection

Colorectal cancer transcriptomic data and clinical annotations were obtained from The Cancer Genome Atlas (TCGA; COAD, and READ projects) for differential expression analysis, pathway exploration, survival assessment, and immune infiltration correlation. Independent validation cohorts were downloaded from the Gene Expression Omnibus (GEO), including GSE31737, GSE37182, and GSE44076. TCGA RNA-seq data were processed and normalized using standard R pipelines (v4.0.3). GEO microarray matrices were normalized according to the platform recommendations; when multiple probes corresponded to the same gene, the probe with the highest average expression was retained. All statistical tests were two-sided, and *P* < 0.05 was considered significant unless otherwise specified.

### 2.2 Pan-cancer and CRC-specific assessment of STC1

SRplot was used to examine STC1 expression across 33 tumor types, including paired (tumor vs. matched normal) and unpaired comparisons (20). GEPIA2 was applied to validate tumor–normal differences and to stratify CRC patients by STC1 expression (median value as cutoff). For GEO datasets, STC1 expression in CRC tissues versus non-tumor controls was compared separately in each cohort, and results were visualized with ggplot2. Group differences were evaluated with Wilcoxon rank-sum tests (unpaired) or Wilcoxon signed-rank tests (paired).

### 2.3 KEGG enrichment analysis

Differentially expressed genes (DEGs) between CRC and normal tissues were identified using limma (for microarray data) or DESeq2 (for RNA-seq data). DEGs were defined as | log_2_FC| > 1 with adjusted *P* < 0.05. GO and KEGG enrichment analyses were performed using ClusterProfiler to determine biological processes and pathways associated with high STC1 expression. To quantify pathway activity in individual samples, GSVA (method = “ssgsea”) was conducted, generating enrichment scores for each pathway. Spearman correlation analysis was used to assess associations between STC1 expression and pathway scores.

### 2.4 Immune microenvironment correlation analysis

Immune infiltration levels in CRC were estimated via the immunedeconv package using the TIMER algorithm. Relative abundances of major immune subsets (B cells, CD4^+^/CD8^+^ T cells, macrophages, NK cells, endothelial cells, etc.) were computed for each TCGA CRC sample. Spearman correlation was then used to evaluate relationships between STC1 expression and immune infiltration scores. In addition, correlations between STC1 and immune-regulatory/immune-checkpoint genes (including ITPRIPL1, SIGLEC15, TIGIT, CD274, HAVCR2, PDCD1, CTLA4, LAG3, PDCD1LG2, IGSF8, and others) were analyzed to infer potential immunosuppressive links.

### 2.5 Cell lines and culture

Human CRC cell lines HCT116 and SW620 and the human monocyte cell line THP-1 were obtained from the Cell Bank of the Chinese Academy of Sciences (Shanghai, China). HCT116 and SW620 were cultured in RPMI-1640 supplemented with 10% fetal bovine serum (FBS), 1% penicillin/streptomycin, and 1% non-essential amino acids. THP-1 cells were maintained in RPMI-1640 containing 20% FBS with the same additives. For macrophage differentiation, suspension THP-1 cells were treated with phorbol 12-myristate 13-acetate (PMA; final concentration, 100 ng/mL) for 48 h under standard culture conditions to induce differentiation into adherent macrophage-like cells. Cells were incubated at 37 °C in a humidified atmosphere with 5% CO2 and routinely tested to confirm absence of mycoplasma contamination.

### 2.6 SiRNA transfection

Cells were seeded into 6-well plates at 1 × 106 cells/well and transfected at approximately 70%–80% confluence. Two independent siRNAs targeting STC1 (siSTC1-1 and siSTC1-2) and a non-targeting control siRNA were used. Transfection was performed according to the manufacturer’s protocol. After 24 h, STC1 knockdown efficiency was examined by qRT-PCR and Western blotting. Successfully transfected cells were harvested for subsequent migration, invasion, EMT, and signaling assays. siRNA sequences were:

- siSTC1-1: 5’-GCAUUCGUCAAAGAGAGCUTT-3’
- siSTC1-2: 5’-GACACAGUCAGCACAAUCATT-3’.

### 2.7 Wound healing assay

Transfected HCT116 and SW620 cells were cultured to ∼90% confluence in 6-well plates. A sterile 200-μl pipette tip was used to generate a straight scratch. Floating cells were removed by gentle PBS washes, and serum-free medium was added to reduce proliferation-driven closure. Images were captured at 0, 24, and 48 h using an inverted microscope. Migration was quantified by measuring the scratch area reduction relative to baseline using ImageJ. Each condition was tested in triplicate.

### 2.8 Cell migration and invasion assay

Cell migration and invasion were evaluated using Transwell chambers (Corning, USA). For migration assays, 2 × 10^5^ cells in serum-free RPMI-1640 were seeded into the upper chamber, while the lower chamber contained RPMI-1640 supplemented with 20% FBS as a chemoattractant. After 48 h, non-migrated cells were removed, and migrated cells on the lower surface were fixed in paraformaldehyde, stained with 0.1% crystal violet, photographed, and counted in multiple random fields. For invasion assays, inserts were coated with Matrigel before seeding, and the remaining steps were identical. All experiments were conducted in three independent replicates.

### 2.9 Quantitative real-time PCR

Samples from the corresponding groups of CRC cells were collected. Total RNA was extracted using a Takara RNA extraction kit, and RNA concentration was determined. Then, reverse transcription was performed using a reverse transcription kit (Takara, Biotechnology) to obtain the target cDNA. Using cDNA as a template, quantitative detection and analysis were conducted via quantitative real-time PCR. The corresponding primer sequences were as follows:

hr18s-F: 5’-AGTCCCTGCCCTTTGTACACA-3’

hr18s-R: 5’-CGATCCGAGGGCCTCACTA-3’

hSTC1-F: 5’-GCAGGAAGAGTGCTACAGCAAG-3’

hSTC1-R: 5’-CATTCCAGCAGGCTTCGGACAA-3’

hIL-10-F: 5’-TGCCTTCAGCAGAGTGAAGA-3’

hIL-10-R: 5’-GGTCTTGGTTCTCAGCTTGG-3’

hTGF-β1-F: 5’-TGGTGGAAACCCACAACGAA-3’

hTGF-β1-R: 5’-GAGCAACACGGGTTCAGGTA-3’

hTGF-β2-F: 5’-AAGAAGCGTGCTTTGGATGCGG-3’

hTGF-β2-R: 5’-ATGCTCCAGCACAGAAGTTGGC-3’

hTGF-β3-F: 5’-CTAAGCGGAATGAGCAGAGGATC-3’

hTGF-β3-R: 5’-TCTCAACAGCCACTCACGCACA-3’.

### 2.10 ELISA assays

Cell culture supernatant was collected, and the secretion levels of TGF-β1, TGF-β2, and TGF-β3 were detected according to the instructions of the corresponding ELISA kit from CUSABIO Biological Engineering Co., Ltd., (Wuhan, China).

### 2.11 Establishment of the co-culture system

For the co-culture system of macrophages and CRC cells, Transwell chambers were used for indirect co-culture. CRC cells were seeded in the lower chamber of 24-well plates (3 × 10^5^ cells/well), and THP-1-derived M2 macrophages were seeded in the upper chamber of 24-well plates (3 × 10^5^ cells/well), with complete 1,640 medium added to both chambers. Control and experimental groups were set as follows: Control group: CRC cells co-cultured with M2 macrophages; Experimental Group: STC1 knockdown CRC cells co-cultured with M2 macrophages. After 48 h of culture, CRC cells were collected for assays to detect the invasive and migratory abilities of TNBC cells.

### 2.12 Flow cytometry analysis

After M0 macrophages were stimulated with CRC cell–conditioned medium to induce polarization, cells were harvested from each treatment group and resuspended in PBS. Macrophages (1 × 10^6^ cells per sample) were incubated with CD11b-PE and CD206-FITC antibodies (BD Pharmingen) at 4 °C for 30 min, followed by flow cytometric analysis.

### 2.13 Western blot (WB) analysis

After cell transfection, total cellular proteins were extracted using a protein extraction kit (Beyotime, China), and protein concentration was determined using a concentration assay kit (Beyotime, China). Proteins were denatured in a dry bath, and protein concentration was measured again before loading. Protein samples were separated by 10% SDS-PAGE gel electrophoresis and transferred to PVDF membranes, which were blocked with 5% BSA. The membranes were then incubated with different primary antibodies at 4 °C overnight. On the next day, the membranes were incubated with the corresponding secondary antibodies at room temperature for 1 h. After washing, the membranes were developed using a high-sensitivity enhanced chemiluminescence reagent (Beyotime, China) on an imaging system (Bio-Rad, United States). Antibodies against E-cadherin (WL01482), N-cadherin (WL01047), Vimentin (WL01960), TGF-β1 (WL02998), P-Smad2/3 (WL02305), and β-actin (WL01372) were purchased from WanLeibio (China). Antibodies against Smad2/3 (5678) and horseradish peroxidase (HRP)-conjugated secondary antibodies (7074) were obtained from Cell Signaling Technology (Massachusetts, United States). Antibodies against Smad4 (230815) and STC1 (124891) were purchased from abcam (Cambridge, United Kingdom).

### 2.14 Immunofluorescence assay

After cell transfection, cells were seeded on cell climbing slides. After the cells adhered naturally, they were washed three times with PBS, fixed with 4% paraformaldehyde, and blocked with 10% goat serum for 30 min. Subsequently, the cells were treated with the corresponding primary and secondary antibodies, and finally, cell nuclei were stained with DAPI. Images were captured under a fluorescence microscope.

### 2.15 Statistical analysis

Experimental data were statistically analyzed using GraphPad Prism 8.0 software. All data were presented as the mean ± standard deviation from at least three independent experiments. Unpaired *t*-tests were used for comparisons between two groups, and one-way analysis of variance (ANOVA) followed by Duncan’s multiple comparison test was used for comparisons among multiple groups. A *P*-value < 0.05 was considered statistically significant.

## 3 Results

### 3.1 STC1 is upregulated in CRC and predicts poor prognosis

**FIGURE 1:**
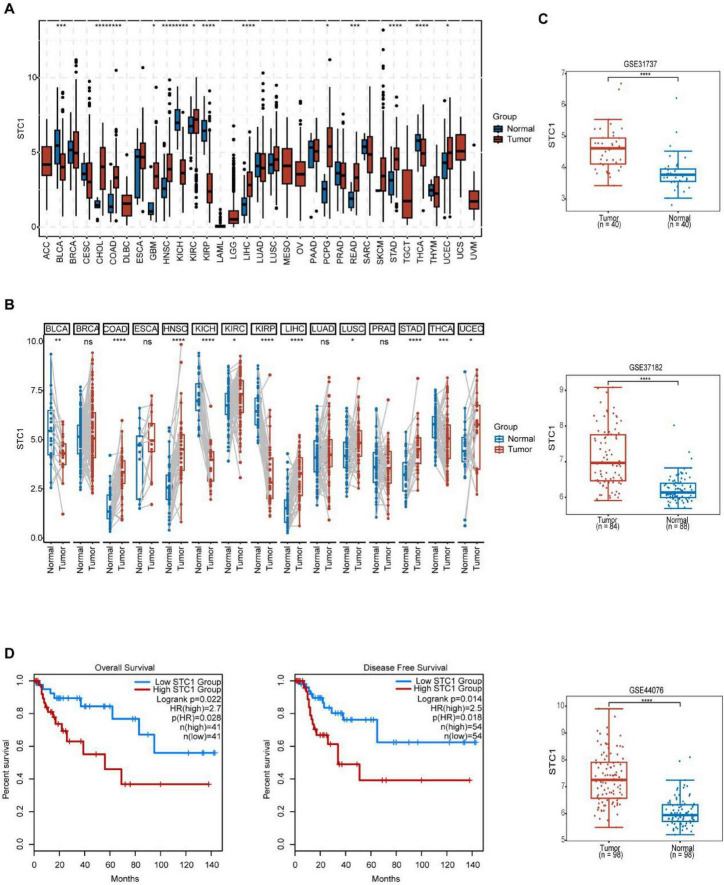
Stanniocalcin-1 (STC1) is upregulated in colorectal cancer (CRC) and predicts poor prognosis. **(A)** Comparison of STC1 expression levels between adjacent normal tissues and tumor tissues in a non-paired manner across various cancer types. **(B)** Comparison of STC1 expression levels between adjacent normal tissues and tumor tissues in a paired manner across various cancer types. **(C)** Comparison of STC1 expression levels between adjacent normal tissues and tumor tissues in colorectal cancer from the Gene Expression Omnibus (GEO) dataset. **(D)** Kaplan-Meier curves depicting the impact of high and low STC1 expression on patient prognosis. *P < 0.05, **P < 0.01, ***P < 0.001, ****P < 0.0001 compared with normal tissues.

To establish the clinical relevance of STC1, pan-cancer profiling was first performed. SRplot analysis across 33 malignancies demonstrated that STC1 expression was significantly elevated in multiple tumor types, including colon adenocarcinoma (COAD) and rectal adenocarcinoma (READ), compared with normal tissues (Figure 1A). Paired tumor-normal comparisons further confirmed consistent STC1 upregulation in CRC specimens (Figure 1B). We then validated these findings in independent GEO CRC cohorts. In GSE31737, GSE37182, and GSE44076, STC1 transcript levels were clearly higher in tumor samples than in non-tumor controls, with concordant trends across datasets (Figure 1C), indicating that STC1 overexpression is reproducible and not cohort-specific. To evaluate prognostic significance, Kaplan–Meier analyses in GEPIA2 revealed that patients with high STC1 expression had significantly shorter OS and DFS than those with low STC1 expression (Figure 1D). Collectively, these data indicate that STC1 is aberrantly upregulated in CRC and may serve as a clinically meaningful risk indicator.

### 3.2 STC1 enhances CRC cell invasion, migration and EMT

**FIGURE 2:**
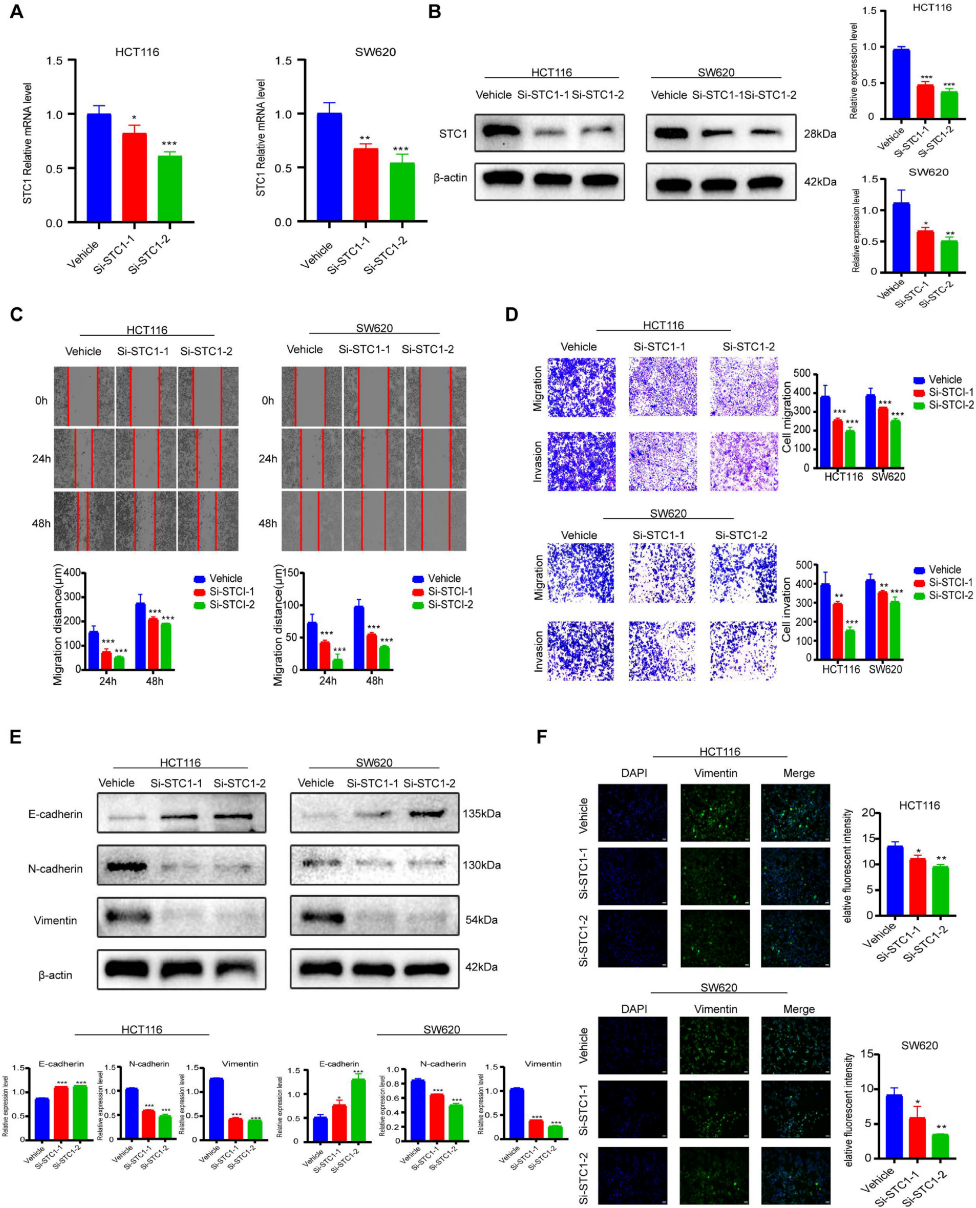
Stanniocalcin-1 (STC1) promotes colorectal cancer (CRC) cell invasion and migration. **(A)** STC1 knockdown efficiency by qRT-PCR. **(B)** STC1 knockdown efficiency by Western blot. **(C)** Scratch healing assay showing the effect of STC1 silencing on cell migration. **(D)** The Transwell assays showing the effect of STC1 knockdown on cell migration and invasion. **(E)** Western blot analysis of E-Cadherin, N-Cadherin, and Vimentin proteins after STC1 knockdown. **(F)** Immunofluorescence staining of vimentin after STC1 knockdown. Scale bar: 200 μm. Data represent three independent experiments. Compared with the control group, *P < 0.05, **P < 0.01, ***P < 0.001.

To investigate whether STC1 functionally contributes to CRC progression, STC1 was silenced in HCT116 and SW620 cells using two independent siRNAs. qRT-PCR demonstrated robust reduction of STC1 mRNA, and Western blotting confirmed marked suppression at the protein level (Figures 2A,B), supporting effective knockdown.

In wound-healing assays, control cells exhibited progressive scratch closure over 24–48 h, whereas both siSTC1 groups showed substantially delayed wound closure (Figure 2C), indicating impaired migratory capacity. Consistently, Transwell assays demonstrated that STC1 depletion significantly reduced the number of migrated cells. In Matrigel-coated inserts, invasive cell counts were also markedly decreased upon STC1 knockdown (Figure 2D). These findings show that STC1 promotes both migration and invasion in CRC cells. Because EMT is a key driver of metastatic dissemination, we examined EMT markers. STC1 knockdown increased the epithelial marker E-cadherin and decreased mesenchymal markers N-cadherin and Vimentin (Figure 2E), indicating EMT suppression. Immunofluorescence staining additionally showed reduced vimentin signal intensity and more epithelial-like cellular morphology in STC1-silenced cells (Figure 2F). Together, these data demonstrate that STC1 is required for maintaining EMT-associated phenotypes and metastatic potential in CRC.

### 3.3 STC1 activates the TGF-β1/Smad signaling pathway in CRC

**FIGURE 3:**
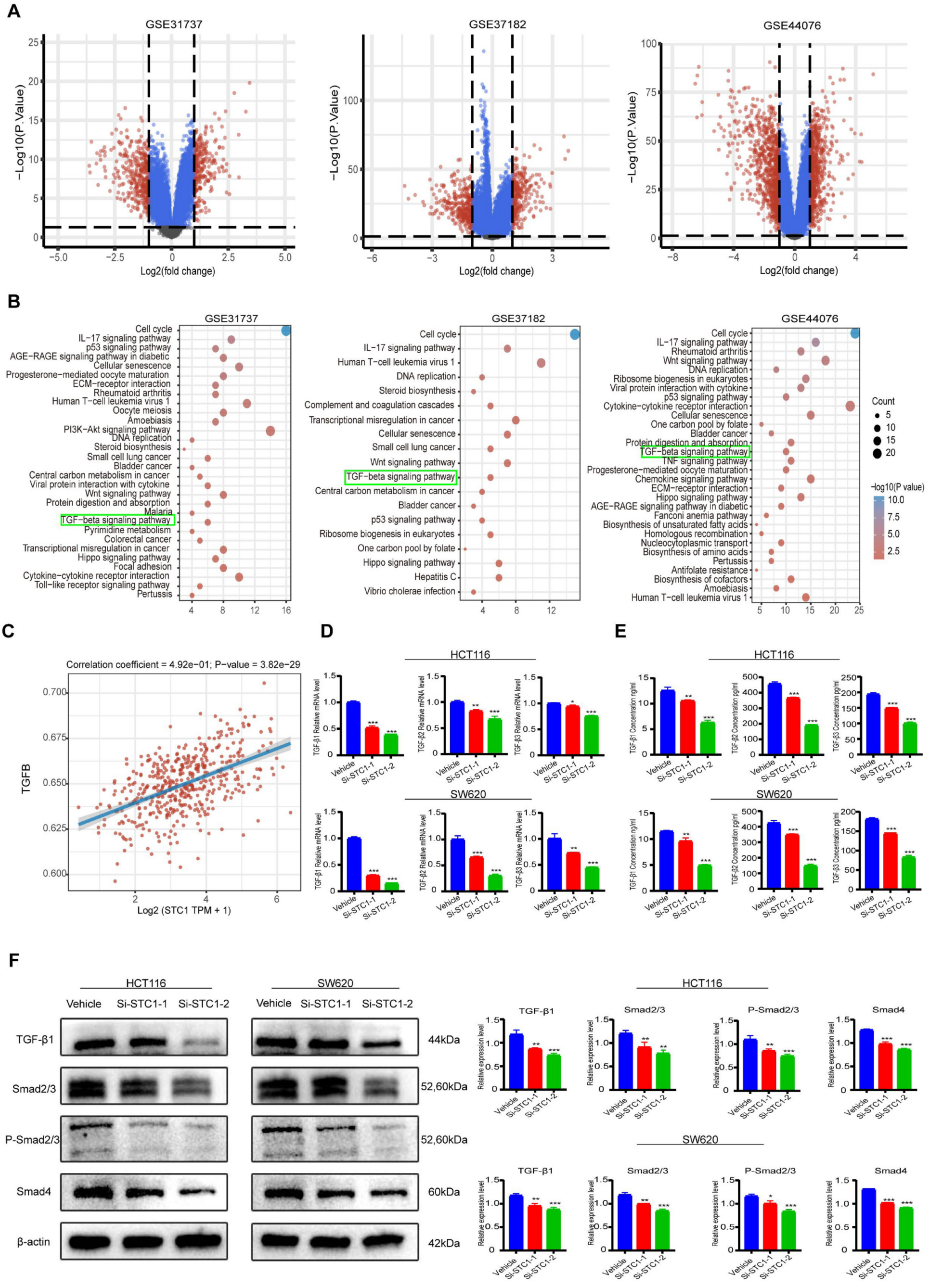
Stanniocalcin-1 (STC1) activates the TGF-β/Smad signaling pathway in colorectal cancer. **(A)** Volcano plot showing differentially expressed genes (DEGs) in the CRC dataset. **(B)** KEGG pathway enrichment analysis of DEGs. **(C)** Correlation between STC1 and the TGF-β pathway. **(D)** qRT–PCR analysis of TGFβ1, TGFβ2, and TGFβ3 mRNA levels in CRC cells after STC1 modulation, with quantification. **(E)** ELISA measurement of secreted TGF-β1, TGF-β2, and TGF-β3 in CRC cells after STC1 modulation, with quantification. **(F)** Western blot analysis of TGF-β1/Smad pathway–related proteins after STC1 modulation, with densitometric quantification. Data represent three independent experiments. Compared with the control group, *P < 0.05, **P < 0.01, ***P < 0.001.

To explore mechanisms underlying STC1-mediated migration, DEGs were identified in GEO datasets and subjected to KEGG enrichment. STC1 was consistently among the upregulated genes and KEGG analysis highlighted significant enrichment of the TGF-β signaling pathway (Figures 3A,B). Spearman correlation analysis further showed a positive association between STC1 expression and TGF-β pathway activation across CRC samples, suggesting functional coupling (Figure 3C).

However, the TGF-β family comprises three isoforms—TGF-β1, TGF-β2, and TGF-β3—which can differ in expression patterns and biological functions (21). Among them, TGF-β1 is widely considered the predominant isoform in the immune system and a central mediator of tumor-associated immunosuppressive signaling, whereas the contributions of TGF-β2 and TGF-β3 are comparatively less clearly defined in many immunological settings (22, 23). In colorectal cancer, published evidence has also linked increased TGF-β1 levels in primary tumors with advanced disease and unfavorable clinical outcomes (24). These observations reinforced the need to clarify whether TGF-β1 represents the major isoform associated with STC1 in our study. To address this, we performed isoform-specific qRT–PCR and ELISA assays, and the results indicated that TGF-β1 showed a closer relationship with CRC samples and STC1-related changes than TGFβ2 or TGFβ3 (Figures 3D,E). Based on this isoform-level evidence and the established biological relevance of TGF-β1 in cancer progression, we next focused on the downstream signaling events most directly linked to migratory phenotypes. In particular, TGF-β1 is known to regulate multiple pathological processes during tumorigenesis and to promote cell migration through the induction of epithelial-mesenchymal transition (EMT) (25, 26). The Smad proteins, core transcription factors of this pathway, play a crucial role in tumor development by regulating the transcription of downstream genes (26). We validated this relationship experimentally. Western blotting showed that STC1 knockdown reduced TGF-β1 levels and attenuated downstream Smad signaling, including decreased total Smad2/3, phosphorylated Smad2/3, and Smad4 (Figure 3F). The coordinated suppression of pathway components indicates that STC1 acts upstream to sustain TGF-β1/Smad activity. Given the established role of TGF-β1 signaling in EMT and migration, these findings provide a mechanistic explanation for the reduced metastatic traits observed after STC1 depletion.

### 3.4 STC1 is associated with an immunosuppressive microenvironment and macrophage infiltration

**FIGURE 4:**
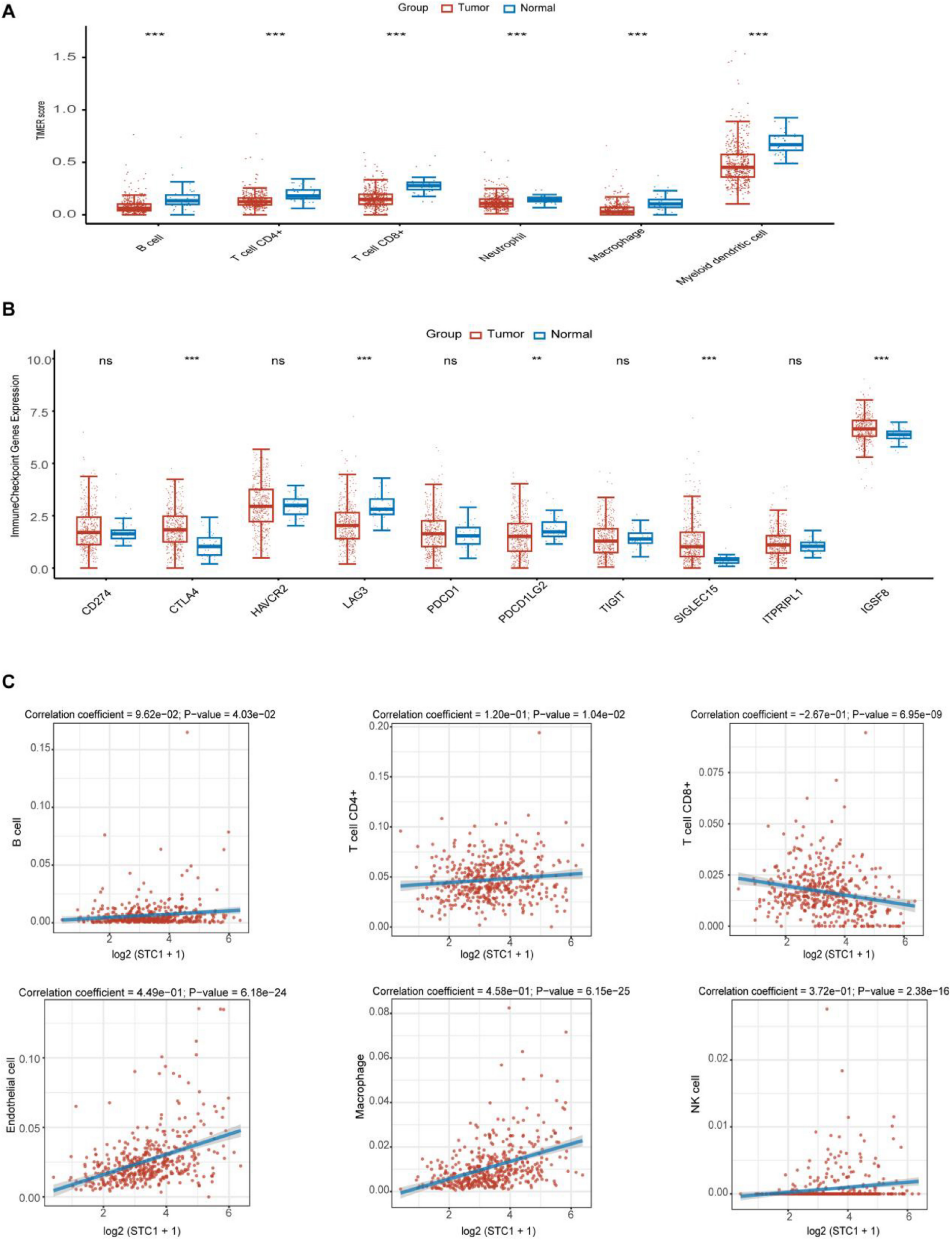
Stanniocalcin-1 (STC1) is associated with immune infiltration in the tumor microenvironment of colorectal cancer. **(A)** Comparison of immune cells in tumor tissues and adjacent normal tissues in colorectal cancer samples. **(B)** Expression distribution of immune checkpoint genes in tumor tissues and adjacent normal tissues in colorectal cancer samples. **(C)** Correlation between STC1 and immune cell score. **P < 0.01, ***P < 0.001.

Based on previous gene enrichment analysis suggesting that STC1 may be involved in the regulation of the colorectal cancer immune microenvironment, we focused on the pro-tumor role of M2-type macrophages in our research. This cell subset drives colorectal cancer progression through mechanisms such as immunosuppression, which has been confirmed in multiple studies. Assessment of immune infiltration characteristics in tumor/paracancerous tissues using the immunedeconv package combined with the TIMER algorithm revealed significant changes in the distribution of six types of immune cells, including macrophages, in the tumor microenvironment (Figure 4A). Given the key regulatory role of immune checkpoint molecules in immune responses, our analysis showed a positive correlation between STC1 expression and the expression of CTLA4, LAG3, PDCD1LG2, SIGLEC15, and IGSF8 (Figure 4B). Spearman correlation analysis further showed that STC1 expression levels were positively correlated with the infiltration of B cells, CD4^+^ T cells, endothelial cells, macrophages, and NK cells, while negatively correlated with CD8^+^ T cells, which play a crucial role in anti-tumor processes (Figure 4C). Collectively, these results indicate that STC1 synergistically induces the expression of immune checkpoint molecules by positively regulating immune components such as macrophages, ultimately inhibiting the efficacy of the body’s anti-tumor immune response.

### 3.5 STC1 induces M2 macrophage polarization and creates a positive feedback loop for CRC migration and invasion

**FIGURE 5:**
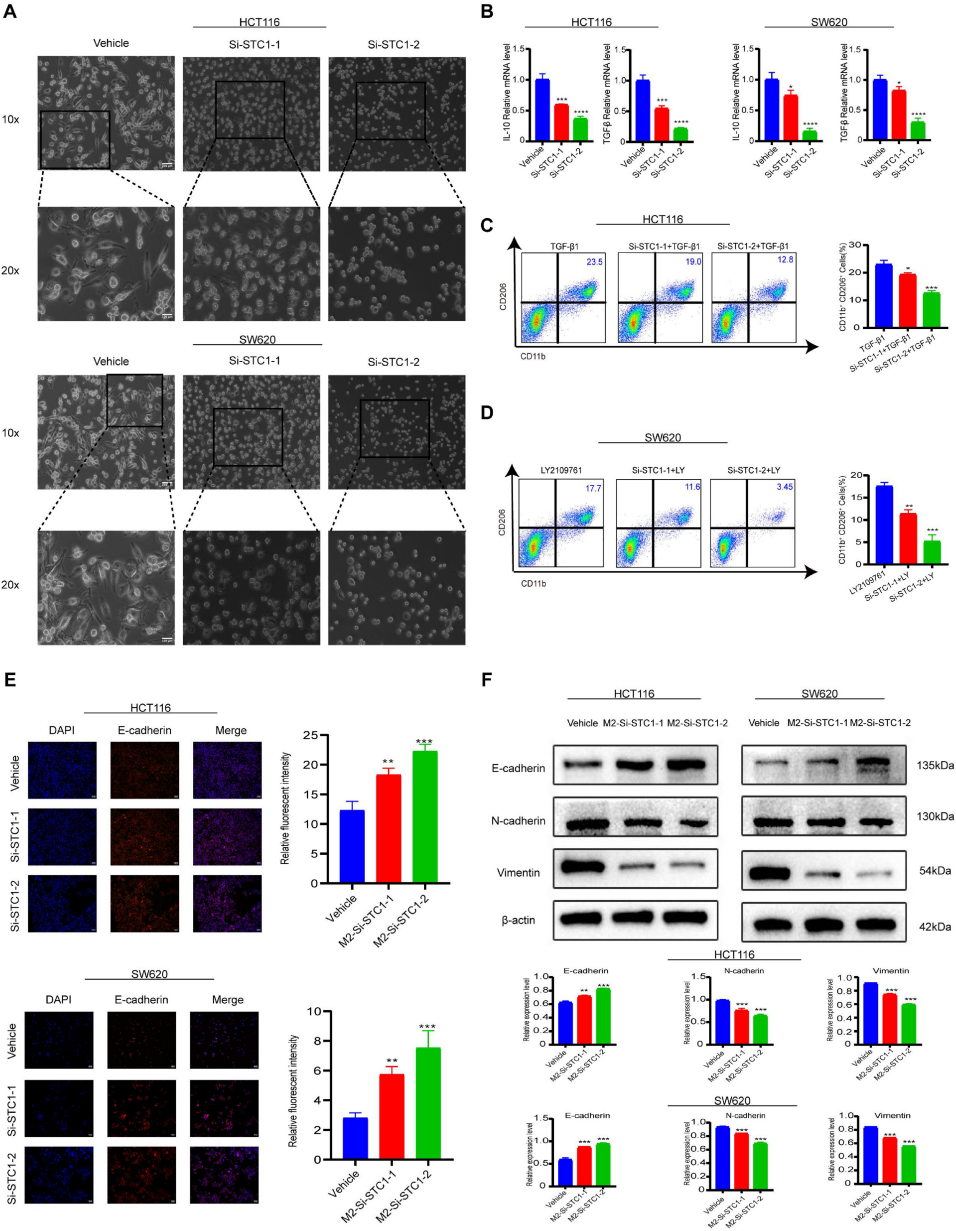
Stanniocalcin-1 (STC1) and M2-type macrophages form a positive feedback loop that enhances colorectal cancer (CRC) invasion and migration. **(A)** Under STC1 knockdown, the Morphological changes of M0 macrophages toward an M2 phenotype was attenuated. Scale bar: 100/200 μm. **(B)** qRT-PCR examination of IL-10 and TGF-β expression in M2-type macrophages. **(C)** Flow cytometry analysis of the proportions of CD11b^+^CD206^+^ cells after treatment with recombinant TGF-β in HCT116 cell–conditioned medium. **(D)** Flow cytometry analysis of the proportions of CD11b^+^CD206^+^ cells after treatment with LY2109761 in SW620 cell–conditioned medium. **(E)** IF examination of E-cadherin protein expression in M2-CRC cells, with quantification. Scale bar: 200 μm. **(F)** Western blot analysis of EMT-related proteins (E-cadherin, N-cadherin, and vimentin) in M2-CRC cells, with densitometric quantification. The data represent three independent experiments. Compared with the control group, *P < 0.05, **P < 0.01, ***P < 0.001, ****P < 0.0001.

Conditioned medium from CRC cells transfected with STC1 knockdown was used to treat M0-type macrophages. Morphological observation showed that macrophages treated with the supernatant from the STC1 knockdown group did not undergo significant morphological changes, while the supernatant from the control group CRC cells significantly induced M2-type macrophage polarization (Figure 5A). qRT-PCR detection further confirmed this phenomenon, as the mRNA expression levels of M2-associated cytokines IL-10 and TGF-β in the STC1 knockdown group were significantly lower than those in the control group (Figure 5B).

Notably, accumulating evidence from previous studies supports a functional role of TGF-β in shaping macrophage polarization toward an M2-like, immunosuppressive phenotype rather than serving merely as a correlated marker. For example, work in THP-1–derived macrophages has shown that exogenous TGF-β stimulation increases IL-10 and M2-associated markers and is accompanied by activation of canonical downstream signaling (27). Complementary findings have further indicated that TGF-β1–Smad signaling contributes to alternative (M2) macrophage activation in conditioned-medium contexts, reinforcing the concept that TGF-β1 can act as an upstream driver of M2-like polarization programs (28). Guided by these observations, we next examined whether TGF-β signaling contributes to STC1-associated macrophage polarization in our system. To this end, we performed flow cytometric analyses of M2 surface markers under TGF-β–related interventions. Upon supplementation with recombinant TGF-β (MCE HY-P7118), the proportion of CD11b^+^CD206^+^ macrophages remained lower in the STC1 knockdown–conditioned groups than in the corresponding control groups in the HCT116 cell model, indicating that STC1 depletion was associated with reduced induction of a TGF-β–linked polarization phenotype in this setting (Figure 5C). Moreover, when the culture system was treated with the TGF-β receptor inhibitor LY2109761 (Selleck S2704), flow cytometry again revealed a decreased proportion of CD11b^+^CD206^+^ cells compared with controls in the SW620 cell model (Figure 5D). Notably, under TGF-β receptor blockade, the reduction in CD11b^+^CD206^+^ cells was more pronounced, supporting the interpretation that inhibition of TGF-β signaling attenuates the capacity of tumor cell–derived factors to drive M2-like polarization, which is consistent with our proposed involvement of the TGF-β pathway.

To evaluate the potential feedback effect of polarized macrophages on CRC cell behavior, we established an indirect Transwell co-culture system between M2-type macrophages and CRC cells. Immunofluorescence detection showed that E-cadherin fluorescence was higher CRC cells from the STC1 knockdown group than in the corresponding control group when co-cultured with M2-type macrophages, indicating a more pronounced restoration of the epithelial marker following STC1 silencing under these conditions (Figure 5E). In parallel, Western blot analysis confirmed corresponding changes in EMT-related proteins (E-cadherin, N-cadherin, and Vimentin) (Figure 5F). Collectively, these results suggest that STC1 in CRC cells is associated with a tumor-macrophage interaction that promotes M2-llike polarization and, in turn, supports pro-migratory and pro-invasive phenotypes in CRC cells, with TGF-β signaling contributing to this crosstalk.

## 4 Discussion

Colorectal cancer is regarded as one of the most prevalent malignant tumors, and the tumor microenvironment plays a crucial regulatory role in the progression of colorectal cancer invasion and migration. Therefore, this study aimed to systematically explore the potential role of STC1 in colorectal cancer and the tumor microenvironment. Our results showed that STC1 is significantly expressed in colorectal cancer and closely associated with M2-type tumor-associated macrophages. Validated through *in vitro* experiments, our findings suggest that STC1 may regulate M2-type macrophage polarization to contribute to colorectal cancer progression.

Stanniocalcin-1 is a glycoprotein hormone involved in calcium-phosphorus homeostasis and has previously been identified as a useful predictor of poor post-operative outcomes in colorectal cancer patients (29). Additionally, STC2, another member of the STC protein family, has been confirmed to promote the proliferation and migration of colorectal cancer cells via the Wnt/β-catenin pathway, participating in tumorigenesis and development (30). However, the regulatory role and molecular mechanism of STC1 in the tumor microenvironment remain poorly understood. We believe that in-depth research on STC1 can provide important insights and offer new ideas for the development of colorectal cancer treatment.

First, through pan-cancer analysis, we systematically analyzed the expression characteristics and clinical prognostic value of STC1, and found that it is specifically highly expressed in colorectal cancer tissues and significantly associated with poor survival outcomes of patients. This conclusion was consistently validated in multiple independent microarray datasets, suggesting that STC1 can serve as a potential prognostic biomarker for colorectal cancer.

Further exploration of its functional mechanism revealed that in the HCT116/SW620 cell models, STC1 knockdown significantly inhibited cell migration and invasion capabilities (decreased migration rate in the scratch wound assay and reduced number of invasive/migratory cells in the Transwell assay). At the molecular level, Western blot and immunofluorescence confirmed that STC1 deficiency could reverse the EMT process, characterized by upregulated expression of the epithelial marker E-cadherin and downregulated expression of the mesenchymal markers N-cadherin and Vimentin. Gene enrichment analysis combined with protein validation revealed that STC1 drives tumor progression-associated phenotypes by activating the TGF-β pathway, an immune-related signaling pathway. To refine this mechanism at the isoform level, we further performed qRT–PCR and ELISA assays to distinguish among the three TGF-β isoforms (TGF-β1, TGF-β2, and TGF-β3), and these analyses consistently indicated that TGF-β1 was the predominant isoform associated with STC1 modulation in our system. Consistent with these findings, Western blotting also supported reduced TGF-β1 levels and attenuation of downstream Smad signaling upon STC1 knockdown, further substantiating involvement of the TGF-β1/Smad signaling pathway. The classic Smad proteins in the TGF-β1 signaling pathway can regulate tumor migration and immune regulation, and TGF-β1 can modulate various immune cells in the TME, including macrophages (31).

Given the impact of tumor-associated macrophages on colorectal cancer progression and treatment, it is particularly important to gain in-depth insight into the role of STC1 in the tumor microenvironment and its potential for immunotherapy. Notably, our results revealed a significant association between STC1 and immune scores, as well as a significant positive correlation with the expression of five classic immune checkpoint molecules, namely CTLA4, LAG3, PDCD1LG2, SIGLEC15, and IGSF8. Moreover, STC1 positively regulates macrophage infiltration. To further clarify the potential signaling basis of this observation, we performed flow cytometry to evaluate M2-associated surface markers under TGF-β–related interventions. Supplementation with recombinant TGF-β1 promoted an M2-like polarization profile in our system, whereas treatment with the TGF-β receptor inhibitor LY2109761 attenuated this polarization tendency. These findings support the involvement of TGF-β signaling in macrophage polarization and are consistent with the notion that STC1 may influence M2-like polarization at least in part through targeting the TGF-β1 axis, although additional *in vivo* validation is still required. Our functional experiments confirmed that STC1 induces M2-type macrophage polarization, and the latter forms an “STC1-TAM-EMT” positive feedback loop to synergistically drive migration by secreting factors such as TGF-β1. Based on the dual role of STC1 in tumor proliferation and immune microenvironment remodeling (32, 33), targeting STC1 is expected to become a new strategy for colorectal cancer immunotherapy.

To our knowledge, this study is the first to comprehensively analyze the role of STC1 in the tumor immune microenvironment of colorectal cancer and to validate its functional relevance using multiple *in vitro* approaches, thereby providing multi-level evidence for the involvement of STC1 in CRC migration and invasion–related phenotypes. Nevertheless, several limitations should be acknowledged. First, our functional evidence is mainly derived from *in vitro* assays of migration and invasion, and thus does not directly demonstrate *in vivo* metastasis, which is a multistep biological process that requires validation in appropriate animal models. Second, although the clinical relevance of STC1 was supported by analyses of public datasets (TCGA/GEO), this study lacks independent validation in an external clinical cohort (e.g., IHC and/or qPCR in patient specimens), and further confirmation in well-annotated clinical samples is warranted. Third, given the heterogeneity of CRC and the diversity of available cell models, we only investigated two cell lines (HCT116 and SW620), which may not fully capture cell line–specific or molecular subtype–specific differences in STC1-associated regulation. Fourth, our mechanistic exploration focused primarily on the TGF-β1/Smad pathway, and it remains unclear whether STC1 influences EMT and tumor–immune interactions through additional mechanisms, such as epigenetic regulation (e.g., histone modifications or DNA methylation), metabolic reprogramming, or crosstalk with other pathways (e.g., PI3K/Akt or NF-κB). Finally, while recent evidence suggests that immune cell subsets such as CD8^+^ T cells can critically modulate CRC progression (34). Our current work mainly evaluated macrophage-related changes and did not systematically assess other immune components within the tumor microenvironment.

In future studies, we will expand our validation to include CRC cell lines representing additional molecular subtypes and incorporate *in vivo* models to evaluate the contribution of STC1 to tumor progression in a more physiologically relevant context. We will also perform validation in independent clinical cohorts to further assess the prognostic value and translational potential of STC1. Mechanistically, we plan to investigate whether STC1 drives downstream signaling via epigenetic mechanisms using approaches such as DNA methylation profiling and chromatin immunoprecipitation, and to identify key signaling nodes under STC1 perturbation using phosphoproteomic analyses. In addition, we aim to establish immunocompetent or humanized mouse models to examine how STC1 affects other immune cell populations beyond macrophages, thereby providing a stronger experimental basis for future development of STC1-related therapeutic strategies in CRC.

## 5 Conclusion

In conclusion, our results suggest that STC1 promotes CRC migration and invasion–associated phenotypes and contributes to an immunosuppressive tumor microenvironment, at least in part through activation of the TGF-β1–Smad signaling pathway and induction of M2-like macrophage polarization. While these findings provide preliminary mechanistic insights into STC1-mediated tumor–macrophage crosstalk, further studies incorporating *in vivo* models and independent clinical cohorts are required to validate the role of STC1 in CRC progression and to assess the translational potential of targeting STC1 as a prognostic biomarker and therapeutic strategy.

## Data Availability

The original contributions presented in this study are included in this article/supplementary material, further inquiries can be directed to the corresponding authors.
