# Correction: STC1 promotes colorectal cancer invasion and migration by regulating M2 macrophage polarization via TGF-β1/Smad signaling pathway

**DOI:** 10.3389/fmed.2026.1881139

**Published:** 2026-07-09

**Authors:** Xin Chen, Youying Lai, Yichen Zhou, Haoyue Wang, Junkai Wen, Yi Zou, Haotian Cui, Wanli Deng, Xinwen Ma, Xueqing Hu, Yanbo Zhang

**Affiliations:** 1 Shuguang Hospital Affiliated to Shanghai University of Traditional Chinese Medicine, Shanghai, China; 2 Department of Medical Oncology, Shuguang Hospital, Shanghai University of Traditional Chinese Medicine, Shanghai, China; 3 Clinical Research Unit, Shuguang Hospital Affiliated to Shanghai University of Traditional Chinese Medicine, Shanghai, China

**Keywords:** colorectal cancer, STC1, macrophages, TGF-β1/Smad, invasion and migration

In the published article, there was an error in the equal contribution statement. Author Youying Lai was erroneously omitted as an equal contributing author. The correct equal contribution statement is:

“Xin Chen and Youying Lai contributed equally to this work and share first authorship.”

The original version of this article has been updated.

